# Variance decomposition of protein profiles from antibody arrays using a longitudinal twin model

**DOI:** 10.1186/1477-5956-9-73

**Published:** 2011-11-17

**Authors:** Bernet S Kato, George Nicholson, Maja Neiman, Mattias Rantalainen, Chris C Holmes, Amy Barrett, Mathias Uhlén, Peter Nilsson, Tim D Spector, Jochen M Schwenk

**Affiliations:** 1 Department of Twin Research and Genetic Epidemiology, King's College London, St. Thomas' Hospital, Westminster Bridge Road, London SE1 7EH, UK; 2 Department of Statistics, University of Oxford, 1 South Parks Road, Oxford OX1 3TG, UK; 3 Science for Life Laboratory Stockholm, KTH - Royal Institute of Technology, Box 1031, SE-171 21 Solna, Sweden; 4 Oxford Centre for Diabetes, Endocrinology and Metabolism, Churchill Hospital, Old Road, Headington, Oxford, OX3 7LJ, UK; 5 Respiratory Epidemiology and Public Health, Imperial College London, Manresa Road, London SW3 6LR, UK

**Keywords:** Variance decomposition, linear mixed-effects model, longitudinal twin study, suspension bead arrays, antibodies, protein profiling

## Abstract

**Background:**

The advent of affinity-based proteomics technologies for global protein profiling provides the prospect of finding new molecular biomarkers for common, multifactorial disorders. The molecular phenotypes obtained from studies on such platforms are driven by multiple sources, including genetic, environmental, and experimental components. In characterizing the contribution of different sources of variation to the measured phenotypes, the aim is to facilitate the design and interpretation of future biomedical studies employing exploratory and multiplexed technologies. Thus, biometrical genetic modelling of twin or other family data can be used to decompose the variation underlying a phenotype into biological and experimental components.

**Results:**

Using antibody suspension bead arrays and antibodies from the Human Protein Atlas, we study unfractionated serum from a longitudinal study on 154 twins. In this study, we provide a detailed description of how the variation in a molecular phenotype in terms of protein profile can be decomposed into familial i.e. genetic and common environmental; individual environmental, short-term biological and experimental components. The results show that across 69 antibodies analyzed in the study, the median proportion of the total variation explained by familial sources is 12% (IQR 1-22%), and the median proportion of the total variation attributable to experimental sources is 63% (IQR 53-72%).

**Conclusion:**

The variability analysis of antibody arrays highlights the importance to consider variability components and their relative contributions when designing and evaluating studies for biomarker discoveries with exploratory, high-throughput and multiplexed methods.

## Introduction

There is an enormous unmet need for biomarkers to characterize disease type, status, progression, and response to therapy. A biomarker, which can be defined as a physical sign or laboratory measurement that serves as an indicator for biological processes, pathogenic processes, or pharmacologic responses to a therapeutic intervention [1,2], can then potentially be used for screening, prognosis, monitoring response to treatment, and detection of recurrent diseases. Technological advances in the different fields of life science have enabled the generation of data from high-throughput screening experiments, which can then be used to identify novel biomarker molecules.

Proteomics, the large-scale study of the protein content of cells, organs or organisms, offers the potential to evaluate global changes in protein expression, protein interaction patterns and their post-translational modifications that take place in response to normal or pathological stimuli. The availability of DNA microarray analysis permits expression of thousands of genes to be monitored simultaneously, but RNA expression has been found to correlate significantly to only one third with the corresponding actual protein content [3]. Therefore the importance of proteomic research cannot be overstated, as proteins within the cell provide a structural and functional framework by producing energy and allowing communication, movement, and reproduction [4].

Recent technological advances in the field of genomics and proteomics have also brought about a new statistical research area, the analysis of data from high-throughput screening experiments. Using protein microarray technology, the profiles of thousands of proteins can be monitored in a high-throughput manner with the aim of selecting a subset of proteins that could be characterised as potential biomarkers creating possibilities for diagnostic, prognostic and disease progression monitoring [5]. When proteomics emerged, scientists hoped that available technologies would help them sift through the proteome of blood and tissues to identify profiles of proteins that indicated the presence of a disease. Researchers could then use these molecular signatures as biomarkers to diagnose diseases in their formative stages and therefore be able to tailor appropriate intervention and treatment. However, one problem is that the most preferred sample types, serum or plasma, are highly complex and influenced by a protein composition where 99% of the total protein mass is covered by only 20 proteins. Even though all proteins are likely to appear in blood at a given point in time, due to function, secretion or leakage, their levels can vary by more than 10 orders of magnitude, with abundant proteins masking the presence of rare ones. Among the proteomic approaches applied in the quest for novel biomarkers today, mass spectrometry, often in combination with 2D-gel electrophoresis or chromatographic separation, is still the most commonly used technique in the field [6], even though the technique is limited with regard to new diagnostics [7]. Affinity-based alternatives are on the rise to enable proteome-wide analysis, and initiatives have been set up to produce, validate and offer a larger number of validated affinity reagents [8]. Platforms such as microarrays provide solutions for the implementation of larger sets of affinity reagents and analysis of multiple parameters on a minute amount of sample within a single experiment [9]. Among the immunoassay platforms that employ directly labelled samples [10-12], antibody suspension bead arrays to perform highly multiplexed protein profiling in a large number of serum samples [13].

### Utility of twin studies

The phenotype of an individual might change as a result of complex, dynamic interactions between an individual's genome and environmental factors. To characterize and quantify the contributions that genes, the shared and the individual-specific environment and the interactions of these make to human complex traits and phenotypes, we use data from relatives who grow up in similar environments but are of differing genetic relatedness (the so-called 'twin design'). For a long time, twin studies [14] have been a valuable source of information about the genetic basis of complex traits. Twins provide a powerful design for inferring genetic effects, as they are blocked for *in utero*, dietary and socio-economic effects due to common upbringing. Identical/monozygotic (MZ) twins derive from a single fertilized egg and therefore inherit identical genetic material while non identical/dizygotic (DZ) twins share 50% of their genetic material. In a classical twin study the assumption is that MZ and DZ twins share the same degree of similarity in their environments and therefore one compares phenotypic concordance within MZ twin pairs to the concordance within DZ twin pairs. Comparing the resemblance of MZ twins for a trait or disease with the resemblance of DZ twins offers the first estimate of the extent to which genetic variation determines phenotypic variation of the trait or disease. The sources of variation commonly considered in genetics are additive genetic influences (sum of individual effects of all loci that influence the phenotype), dominant genetic influences (these represent interactions between alleles at all loci that influence the phenotype), common environment (influences shared by family members e.g. socio-economic status) and individual environment (influences that cause differences among members of one family, e.g. dietary and lifestyle choices that are not shared by other family members. Two important population parameters are heritability and familiality. The narrow-sense heritability is the proportion of phenotypic variation within a population attributable to additive genetic effects. Familiality is the proportion of phenotypic variation within a population attributable to genetic (additive and dominant) and common environmental effects.

In the following, we have investigated the variance of exploratory affinity arrays using longitudinal twin study.

## Results

Important attributes of discovered biomarkers, in addition to being associated with a disease, are that they can be measured with precision (low variance) and exhibit relatively low amounts of short-term variability relative to the effect size of the difference between healthy and diseased individuals [15]. For antibody-based proteomics, antibodies that have high proportions of variability attributable to familiality and individual environment and have relatively small technical variability are therefore potentially more valuable for use in biomarker discovery.

### Pre-processing

**Figure 1:**
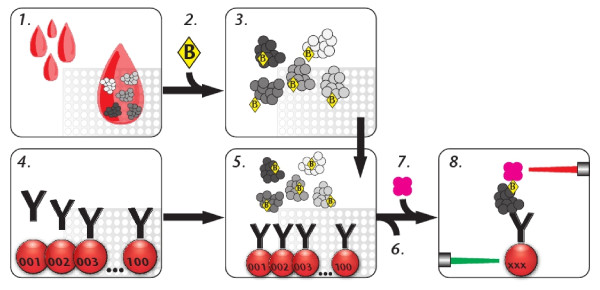
**Experimental workflow** - The process begins with (1.) the distribution of samples into microtiter plates according to a defined layout, dilution and heat treatment. (2.) The protein content of the samples is then label with biotin and (3.) the samples are then prepared for the assay and heat-treated again. Alongside this, (4.) the antibodies are coupled onto beads with distinct color-codes and an array in suspension is created and (5.) beads and samples are combined and incubated. (6.) Proteins that have not been captures by the antibodies are removed and (7.) fluorescent streptavidin is added to bind to the target proteins via their biotin modification. (8.) The beads are then measured and the co-occurrence of beads, which are identified via a green laser, and the emitted reporter fluorescence, excited by a red laser, allowing determining interaction dependent intensity values in multiplex.

For this study the measured relative quantity of interest is the intensity of fluorescence emitted and measured when antibodies immobilized onto beads capture their target protein from a sample (see Figure 1). The intensity levels are influenced by a number of underlying factors besides the antigen's abundance. These are antibody-antigen kinetics and affinities, the number of accessible binding sites on both immobilized antibodies and the labelled antigens, the number of biotin molecules per antigen being incorporated during labelling reaction, as well as complex formation introducing additional biotin molecules carried by proteins attached to the antigen of interest. Therefore the variation in the measured intensity levels will be influenced by both biological and experimental factors including sample preparation, incubation and read-out.

**Figure 2:**
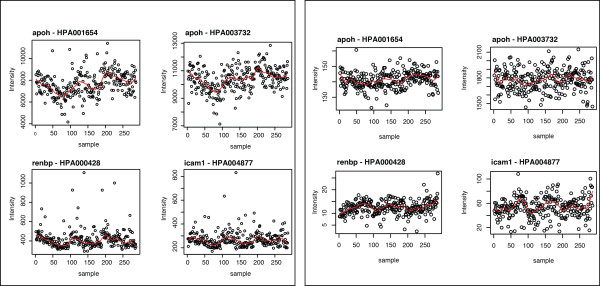
**Protein profiles**. A) Profiles before any data processing are shown from four antibodies across all samples. B) Profiles for the antibodies are shown after normalization, removal of outliers and BoxCox transformation. The red line indicates the locally weighted scatterplot smoothing (LOWESS). The vertical dashed lines differentiate between three microtiter plates in which the samples were distributed. Graphs from all antibodies pre and post data treatment are found in Additional Files 1 and 2.

Molecular phenotypes can be noisy, context dependent, and sensitive to laboratory, equipment and experimental conditions. On the current platform, the fluorescence intensities obtained on each sample can in general not be compared directly due to variations in sample treatment, labelling and detection, thus appropriate pre-processing is needed. Plots of the signal intensities of each antibody across the randomized samples (Figure 2A and Additional File 1) show that for some of the antibodies, such as for example apoh-HPA001654, renbp-HPA000428, apoh-HPA003732 and icam1-HPA004877, signal intensities tended to decrease as intensities were measured from plate well 1 through plate well 96 suggesting an 'intensity-drift effect'. This could be introduced during sample preparation, modification of the potential binding site during sample labelling, slight bleaching of the fluorophore of the reporter protein, or by the dissociation of antibody-antigen complexes over time the samples are being measured.

**Figure 3:**
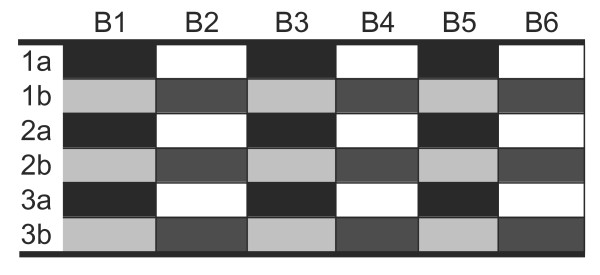
**Correlation model**. Suppose we have three duplicated samples 1, 2 and 3 assayed at 6 antibodies B1 - B6. Denoting the aliquot pairs of the samples as 1a, 1b, 2a, 2b, 3a and 3c (see table above), to check the within sample concordance we look at correlations between the 6 pairs of 3 dimensional vectors. That is for each of the antibodies B1, B2 and B3, we determine the correlation between the black and light-grey vectors. Similarly for antibodies B2, B4 and B6 we look at the correlation between the white and dark-grey vectors.

The data were normalized using probabilistic quotient normalization (PQN)[16]. To assess the efficacy of the normalization we looked at the within-sample concordance before and after normalization, by computing the Spearman correlation between the 48 pairs of aliquot replicates for each antibody in the data set. See appendix for details on the correlation model and an example in Figure 3. Across the 69 antibodies, before normalization the median Spearman correlation was 0.26 with interquartile range (IQR 0.19 - 0.36) and after normalization, median Spearman correlation was 0.36 (IQR 0.24 - 0.44). These results show that normalization improves the within-sample concordance.

### Outliers

**Figure 4:**
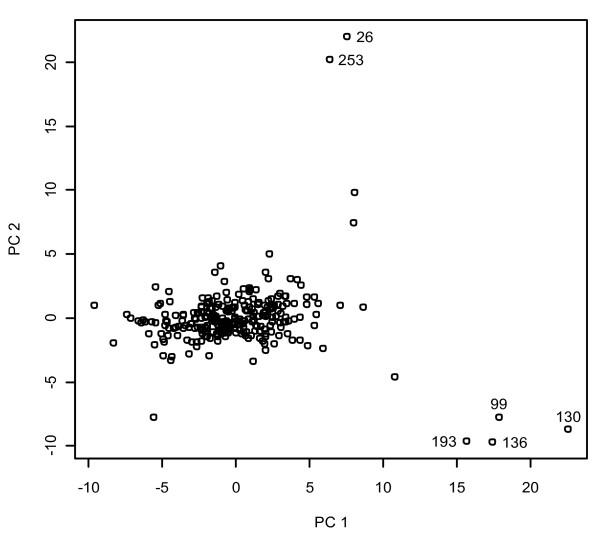
**Principal component projection**. The graph shows the normalized data for the 270 samples in the dataset in the two-dimensional antibodies space. Outlying samples are marked with numbers.

Using singular value decomposition (SVD) [17] we projected the 69 dimensional antibody space into a two-dimensional space (two principal components). Prior to performing SVD on the data matrix, we scaled the data of each variable (antibody) to have a mean of 0 and standard deviation 1. Plots of the two principal components are shown in Figure 4. As can be seen in the figure, most of the samples cluster together. However, there are some outlying samples. For each of the sample points in the figures, Mahalanobis [18] distance from the origin was calculated. Assuming that the principal components have a bivariate normal distribution, the Mahalanobis distance of each sample was compared to a chi-squared distribution with 2 degrees of freedom. A sample was defined as an outlier if it had a Mahalanobis distance bigger than 18.42 (critical value at a 0.0001 level of significance). Based on the above criteria, the data set contains 6 out of 270 samples classified as outliers as shown in Figure 4 highlighted using numbers.

### Variance decomposition

Plots of the data after normalization, removal of outliers and Box-Cox transformations are presented in Figure 2B and Additional File 2. Further, table 1 shows for each antibody the AIC obtained from fitting models 1 and 2 (see Modelling and Estimation in the Materials and Methods Section) on these data. As can be seen in the table, for some antibodies model 1 had a better fit (smaller AIC), whilst for others model 2 had a better fit. This shows that for some antibodies including a linear plate specific intensity-drift effect modelled the data better. Subsequently for each antibody, model 1 or 2 (depending on the model that had a smaller AIC) was used to obtain maximum likelihood estimates of the fixed effects and the variance components.

**Table 1:** Akaike's information criterion

Antibody ID	Gene ID	Gene Name	Model 1	Model 2
HPA002550	ENSG00000118271	ttr	1940	1940
HPA000951	ENSG00000204359	cfb	1047	1047
HPA000952	ENSG00000204359	cfb	758	753
HPA001465	ENSG00000112936	c7	-5645	-5639
HPA003825	ENSG00000131187	f12	-3249	-3248
HPA002350	ENSG00000173372	c1qa	11118	11120
HPA000440	ENSG00000081237	ptprc	-1913	-1910
HPA001816	ENSG00000117601	serpinc1	-3394	-3393
HPA001527	ENSG00000091513	tf	-1849	-1861
HPA001560	ENSG00000114200	bche	-5941	-5938
HPA001654	ENSG00000091583	apoh	396	389
HPA001804	ENSG00000124491	f13a	-9394	-9396
HPA001817	ENSG00000204359	cfb	-2887	-2893
HPA001832	ENSG00000204359	cfb	-4029	-4033
HPA003980	ENSG00000150782	il18	-3202	-3209
HPA004056	ENSG00000091136	lamb1	1311	1285
HPA001833	ENSG00000012223	ltf	-1671	-1676
HPA004061	ENSG00000108666	c17orf75	-4030	-4033
HPA001834	ENSG00000047457	cp	-4851	-4846
HPA001885	ENSG00000167711	serpinf2	3281	3282
HPA001886	ENSG00000168811	il12a	2147	2152
HPA001900	ENSG00000171564	fgb	-2148	-2147
HPA001901	ENSG00000171564	fgb	-12737	-12733
HPA004063	ENSG00000110711	aip	-1996	-1995
HPA004146	ENSG00000080618	cpb2	-1362	-1358
HPA004252	ENSG00000010610	cd4	-1815	-1814
HPA004335	ENSG00000014257	acpp	-1148	-1159
HPA004716	ENSG00000068796	kif2a	-143	-147
HPA004732	ENSG00000178772	cpn2	12220	12217
HPA004796	ENSG00000028137	tnfrsf1b	1555	1541
HPA002265	ENSG00000175899	a2m	1810	1811
HPA004824	ENSG00000049239	h6pd	-1010	-1020
HPA004827	ENSG00000058056	usp13	-1920	-1933
HPA005448	ENSG00000152942	rad17	503	499
HPA005692	ENSG00000091513	tf	1236	1230
HPA006514	ENSG00000143543	jtb	894	895
HPA006493	ENSG00000065833	me1	-2020	-2022
HPA007724	ENSG00000184500	pros1	-1710	-1706
HPA007838	ENSG00000143157	pogk	-3212	-3207
HPA007845	ENSG00000143318	casq1	-4097	-4094
HPA001254	ENSG00000166165	ckb	-5650	-5655
HPA002891	ENSG00000142208	akt1	-1947	-1949
HPA000288	ENSG00000130234	ace2	-3830	-3833
HPA000428	ENSG00000102032	renbp	-5983	-6028
HPA000834	ENSG00000160211	g6pd	-1733	-1733
HPA000572	ENSG00000120885	apoj	-8838	-8845
HPA000793	ENSG00000111674	eno3	-6337	-6336
HPA003732	ENSG00000091583	apoh	3288	3290
HPA001252	ENSG00000150907	foxo1	-5775	-5791
HPA001352	ENSG00000110244	apoa4	799	791
HPA002549	ENSG00000110244	apoa4	4173	4166
HPA001247	ENSG00000179091	cyc1	-4411	-4409
HPA001249	ENSG00000104365	ikbkb	-1732	-1745
HPA004877	ENSG00000090339	icam1	-7497	-7519
HPA010525	ENSG00000163736	gpr1	-4672	-4690
HPA001610	ENSG00000183671	pon1	3271	3274
HPA009656	ENSG00000005421	znf174	-814	-815
HPA002989	ENSG00000103343	sparc	-1962	-1961
HPA003020	ENSG00000113140	sparc	5458	5451
HPA003827	ENSG00000113140	f13b	-1918	-1919
HPA003412	ENSG00000143278	plat	-2180	-2175
HPA007875	ENSG00000104368	mmp3	-1768	-1768
HPA008257	ENSG00000069702	tgfbr3	-7226	-7235
HPA006279	ENSG00000143382	adamtsl4	4013	4004

The Akaike's information criterions (AICs) were obtained for HPA antibodies from fitting models 1 and 2.

For each antibody the percentage of total variance attributable to familiality (fam), individual environment (env), common visit (cv), individual visit (iv) and residual experimental effects (exp) each antibody is presented in Table 2 (rows are ordered by decreasing familiality). In Table 2, we observe that for many proteins the variances of the factors other than experimental variance are estimated to be zero. In general, the maximum likelihood (ML) estimators of the variance components often lie on the boundary of the parameter space i.e. close to zero. This can result from (a) the underlying "true" values of the variances being relatively close to zero and/or (b) there being less information about these variance components, implying that the ML estimators have relatively high variance.

**Table 2:** Phenotypic variance

Antibody ID	Gene Name	fam	env	cv	iv	exp
**HPA001886**	**il12a**	**42.1**	**20.1**	**0**	**0**	**37.8**
**HPA003412**	**plat**	**36.9**	**17**	**0**	**8.4**	**37.7**
HPA002550	ttr	31.3	0.7	0	18	49.9
HPA001816	serpinc1	31	0	2.4	0	66.7
**HPA006279**	**adamtsl4**	**29.7**	**27**	**0**	**0**	**43.3**
HPA002265	a2m	27.9	3	0	20.6	48.5
HPA002549	apoa4	27.4	0	2.2	25.1	45.4
HPA000440	ptprc	26.4	0	0	0	73.6
HPA001817	cfb	26.3	6.4	0	4.2	63.1
HPA007875	mmp3	25.6	0	8	0	66.4
HPA002891	akt1	25.6	0	0	0	74.4
HPA004063	aip	25.5	0	0	8.3	66.1
HPA003732	apoh	25.5	0	4.5	0	70
HPA001352	apoa4	25.2	0	3	31.7	40.2
HPA001610	pon1	24.5	0	0	12.2	63.3
HPA002350	c1qa	24	0	0	9.8	66.2
HPA001249	ikbkb	22.5	0.7	0	24	52.8
HPA001832	cfb	22.5	0	0	19	58.6
HPA005692	tf	22.4	9.1	5	0	63.5
HPA000951	cfb	21.6	19.8	8.2	0	50.5
HPA000952	cfb	21.4	11.9	0.2	9.1	57.4
HPA001900	fgb	20.6	0.5	0	15.8	63.1
HPA001885	serpinf2	20.6	1.4	0	22	56
HPA001247	cyc1	18.8	26.7	0	3.7	50.7
HPA008255	slc27a1	18.5	2.5	0	10.6	68.4
HPA001465	c7	17.9	0	13.2	14.1	54.9
HPA000428	renbp	16.9	17.2	0	9.9	56
HPA009656	znf174	16.1	20.2	0.5	24.3	38.9
HPA004061	c17orf75	15.3	9.8	20.3	0	54.7
HPA007845	casq1	13.9	0	0	0	86.1
HPA006514	jtb	13.6	19.9	0	0	66.5
HPA004732	cpn2	12.9	0	0	0	87.1
HPA001654	apoh	12.2	0	1	5.2	81.6
HPA003020	sparc	11.9	5.6	12.3	34.5	35.5
HPA004716	kif2a	11.3	0	0	0	88.7
HPA004335	acpp	11.3	0	0	0	88.7
HPA002989	sparc	10.9	26.5	0	3.1	59.5
HPA000834	g6pd	10.1	30.9	0	19.1	39.9
HPA001834	cp	9.9	22.4	0	0	67.7
HPA001252	foxo1	7.8	22.2	3.2	0	66.9
HPA008354	ppbp	7.6	9.2	8.5	7.3	67.4
HPA003980	il18	7.3	5.1	17.2	34.4	36
HPA004252	cd4	6.7	3.2	6.6	15.6	67.9
HPA001901	fgb	6.3	7.9	0	19.2	66.6
HPA001527	tf	5.9	8.6	8.9	0	76.7
**HPA008257**	**tgfbr3**	**3.6**	**49.2**	**2.1**	**0**	**45.1**
HPA006493	me1	2.9	1.4	21.2	5.5	69.1
HPA007724	pros1	1.3	21.8	0	20.7	56.2
HPA004877	icam1	1.3	27.1	0	0	71.6
HPA001254	ckb	1.1	21.1	6.5	29.6	41.7
HPA001560	bche	0.9	30.3	8.8	0	60.1
HPA003825	f12	0.5	0	1.6	21.9	76
HPA004824	h6pd	0.1	9.4	0	0	90.6
HPA000793	eno3	0	19.8	2.7	27.8	49.7
HPA005448	rad17	0	2.1	3.9	0	93.9
HPA010525	gpr1	0	11.9	8.3	0	79.9
**HPA000288**	**ace2**	**0**	**58.9**	**2.7**	**6.9**	**31.5**
HPA007838	pogk	0	0	0	31.8	68.2
HPA001804	f13a	0	39.1	0	0	60.9
HPA001833	ltf	0	21.6	0	0	78.4
HPA004146	cpb2	0	0	0	0	100
HPA000572	apoj	0	38.9	0	8.6	52.5
HPA004056	lamb1	0	0	0	40.1	59.9
HPA004796	tnfrsf1b	0	9.4	0	6.7	83.9
HPA004827	usp13	0	0	0	0	100
HPA003827	f13b	0	16.7	8.6	0	74.7

The percentages of phenotypic variance attributable to familial sources (fam), individual environment effects (env), common visit effects (cv), individual visit effects (iv), and experimental effects (exp) are listed for all HPA antibodies. Those HPA antibodies with stable biological variance in excess of 50% are highlighted in bold.

**Figure 5:**
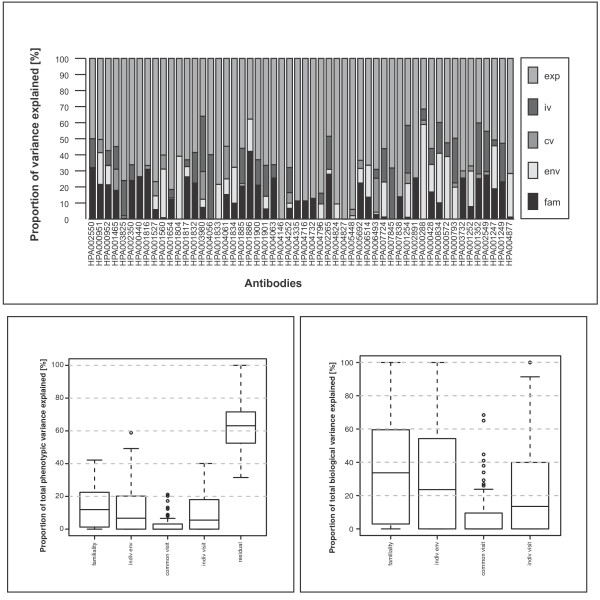
**Variance decomposition**. A) The bar plot summarizes the decomposition of total phenotypic variance of each antibody. The colours in each bar represent the proportion of total phenotypic variability attributable to familiality (fam), individual environment (env), common visit (cv), individual visit (iv), and residual variance (exp). B) Boxplots summarizing the decomposition of total phenotypic variance across all antibodies. C) Boxplots summarizing the decomposition of biological (i.e non- experimental) variability across all antibodies.

Figure 5A represents bar plots of the decomposition of the total phenotypic variance for each antibody after normalization, removal of outliers and Box-Cox transformations. The graph in Figure 5B summarizes the decomposition of total phenotypic variance across all antibodies. As can be seen from these figures, most of the antibody-derived intensities have a high residual (experimental) variance suggesting that a lot of the variance is attributable to experimental sources and sample complexity. Across the 69 antibodies, the median proportion of total phenotypic variance attributable to familiality is 12 (IQR 1-22%); the median proportion of total phenotypic variance attributable to experimental sources is 63% (IQR 53-72%). Combining familiality and individual environment renders the proportion of non-experimental variance that is stable over the time period between the two visits during which data was collected. Across, the 69 antibodies, the median proportion of total variance attributable to familiality and individual environment is 25% (IQR 14-33%). The median proportions of total phenotypic variance attributable to individual visit and common visit is 6% (IQR 0-18%) and 0% (IQR 0-3%), respectively.

In what follows, we present summaries of the proportion of non-experimental variance attributable to familiality, individual environment, common visit and individual visit. The boxplots in Figure 5C summarize the decomposition of non-experimental variance across all antibodies. Across the 69 antibodies the median proportion of non-experimental variance attributable to familial sources is 34% with (IQR 3-60%).. Across all 69 antibodies, the median proportion of non-experimental variance attributable to familiality and individual environment is 71% (IQR 51-93%). The median proportions of non-experimental variance attributable to common visit and individual visit are 0% (IQR 0-10%) and 16% (IQR 0-40%), respectively, indicating that most of the unstable (short-term dynamic) biological variation is attributable to individual visit effects.

### Antibodies for targeting potential biomarkers

**Figure 6:**
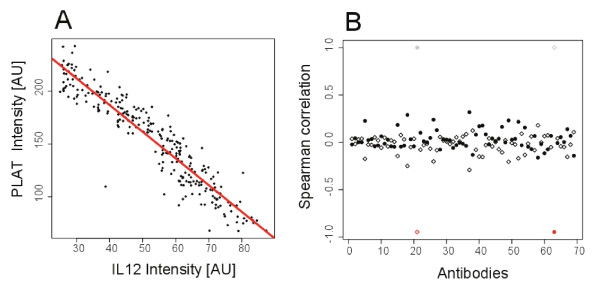
**Correlation of profiles of HPA001886 and HPA003412**. A) The intensity profiles from antibodies targeting IL-12 and PLAT were found to be strongly negatively correlated (R = -0.95, red line), which suggested a biological connection between these two proteins. B) When the correlation investigation of IL-12 (black circles) and PLAT (open diamonds) was extended to all other antibodies, no correlation value outside the range of 0.5 and -0.5 was found.

Highlighting those HPA antibodies with most preferred characteristics, we identified five interesting antibodies where more than 50% of the total phenotypic variance is attributable to stable biological (familial and individual environmental) variability. Among these target proteins were interleukin 12 (il12a), a heterodimeric 70 kDa glycoprotein with implications in cell-mediated immunity [19] targeted by HPA001886 (62%), and angiotensin-converting enzyme 2 (ace2) targeted by HPA000288 (59%). Ace2 is an exopeptidase that catalyses the conversion of angiotensin I or II and its pharmacological inhibition is associated with protective effects from cardiovascular diseases. Further, ADAMTS-like protein 4 (adamstsl4) targeted by HPA006279 (57%) is a secreted glycoprotein with a suggested function to be involved in cell adhesion and protease regulation [20]. The tissue-type plasminogen activator (plat) targeted by HPA003412 (54%) is a secreted serine protease which converts the proenzyme plasminogen to plasmin and up-regulated gene expression has been associated with heart failure [21]. The transforming growth factor beta receptor III (tgfbr3) targeted by HPA008257 (53%) is a glycosylated protein that is found as membrane-bound and as soluble form, and is among the most widely distributed TGF-beta family members and is suggested to function as accessory molecules in TGF-beta and FGF systems [22]. Interestingly, a strong negative correlation between profiles of interleukin 12 and tissue-type plasminogen activator was observed (Figure 6). A linkage between these two proteins has been reported before [23] with il12a inducing the activation of fibrinolysis and thrombin generation. It was also shown that plasma levels of plat decrease shortly after il12 administration but increased over time as il12a levels lowered.

The results suggest that some antibodies are more suitable and promising than others and that they can be potential candidates to be used further in biomarker discovery studies. Among these, HPA006279 has been shown to reveal interesting differences in plasma protein profiles in Metabolic syndrome cases and controls [24]. The identified antibodies should now be further investigated (i) separately, in combination or supplemented by other candidates in high-throughput screenings across diseases, and (ii) to verify their potential implications in diseases related cardiovascular disorders by meeting the criteria of clinical test systems (sandwich assays), which will most likely the improve precision (reduce variance).

## Discussion

In this paper we use a linear mixed-effects model in a unique twin design with duplicated repeated measurements to apportion the total variation of molecular phenotypes (protein profiles) into biological and experimental (technical) variation. Understanding the sources of variation (e.g familial, individual environmental, and experimental) inherent in the measurement of a molecular phenotype is a key step in assessing the potential for stable, informative biomarkers. We observed that across the 69 antibodies the median proportion of total variation attributable to familial sources was 12%. This familial component is consistent with protein profiles having the potential to reflect the polygenic basis of complex disease. Further, across the 66 HPA antibodies, the median proportion of total variation explained by stable sources (familiality and individual environment) was 25%. Stable variation in the current setting comprises that a protein profile remains constant within an individual over the course of the sampling period. A small proportion of variation originated from the short-term biological component, individual visit (median proportion 6%). Common visit represented an inconsiderable amount of variation. Most of the variation originated from experimental sources (median proportion 63%). To our current knowledge, the present study is the first to address the key issue of investigating sources of variation in data generated by exploratory antibody microarrays. It should provide important information when aiming at designing and utilizing such assays and be valuable for multiplexed and quality controlled assays [25] that will become more widely used and accepted for clinical testing.

Ultimately, diagnostic tools built on markers discovered via these screening approaches could become valuable approaches to predict disease state and progression. As an example, antibodies that allow the comparison of individuals that are discordant for diabetes and diabetes-related clinical traits would be useful for identifying individuals likely to suffer from diabetes in the future, long before conventional diagnostic techniques can prove effective. In this way these affinity-based proteomics discoveries would become useful in clinical settings.

However, most antibodies used in this screening method have a large residual variance suggesting that a large proportion of variation in the data is experimentally derived. Potential (and non-separable) sources of this experimental variation, which exclude sample collection, preparation and storage, are the:

(i) complexity and composition of the serum samples which has an effect on the assays;

(ii) biotin modification of samples with regard to the numbers and variability of modifications introduced per molecule and sample;

(iii) sample treatment in terms of liquid transfers, heating, and assay buffer dilution;

(iv) assay procedure with immobilized antibodies selectively capturing aggregated or free molecules from the surrounding solution;

(v) fluorescence-based read-out being influenced by bleaching and dye incorporation onto the target molecules.

(vi) specificity of antibody binding events.

Addressing these issues would lead to a reduction in the proportion of phenotypic variation arising from experimental sources. This would in turn reduce the sample sizes (or degree of technical replication) required to detect epidemiological effects of interest. A reduction of the experimental variability, possible to achieve by using two antibodies to detect a target protein, would ensure that the experimental noise does not swamp biological signals of interest. The technical precision of such a measurement and of other antibodies can be improved, either by addressing the issues outlined above, or, in the short term, by assaying samples in technical replicate.

Research in the field of proteomics is advancing, with affinity-based approaches emerging alongside classical mass spectrometric approaches. With array-based proteomics becoming a promising area in the field of biomedical research, decomposition of the underlying variation in protein profiles into biological (both stable and longitudinally fluctuating) and experimental components is an important and useful step in exploring the applicability of antibody arrays for the exploration of the proteome. Ultimately, such proteomic strategies may lead to new disease markers and drug targets can be identified, benefitting from the possibilities offered by the versatility of both the employed affinity reagents and multiplexed techniques.

## Materials and methods

In this paper we longitudinally collect serum samples from a cohort of 77 twins (56 MZ pairs and 21 DZ pairs) to explore the sources of variation underlying antibody array-based protein phenotypes with the aim of aiding the design and interpretation of future proteomic research. We use a mixed-effects modelling approach to obtain estimates of variance components.

This is a longitudinal family study whose design involves duplicate measurements over time in a sample that includes related individuals (twins). Consequently it combines the features of a longitudinal study (the power to study the pattern of changes in a trait over time) with the features of a cross-sectional twin study (which allows one to estimate the variation in a trait attributable to familial sources).). The study design allows us to break down the observed molecular phenotypic variance into four biological components. These are *familiality *(the combined effects of genetic and common environment) and *individual environment*, both of which are longitudinally stable biological components. The two short-term biological components are *common visit *and *individual visit*, which respectively measure the amount of shared (by twins within a pair) and non-shared longitudinal variation observed over the sampling period of the present study (i.e several months).

### Subject recruitment

A total of 154 healthy, postmenopausal, female twins, comprising of 56 MZ and 21 DZ pairs, were ascertained from the TwinsUK database at St. Thomas' Hospital http://www.twinsUK.ac.uk, and invited to participate in this study. Recruitment of the twins was as follows: eligible twins were sent an information sheet containing details of the study as well as two consent forms. If the twins agreed to participate they signed and sent one copy of their consent forms to the administration team at the Department of Twin Research and genetic epidemiology (DTR), King's College London. Once a consent form was received, a member of the administration team would contact the twins by letter and phone to book their appointment. In addition, 68 twins (34 MZ pairs) were asked to re-attend once they had successfully completed the first visit with the time between the two visits ranging from 63 to 99 days (26 twins), 104 to 140 days (34 twins) and 150 to 216 days (8 twins). At each visit to the clinic, the twins were in pairs.

Fasting blood samples were collected from all selected twins from one or both visits rendering a total of 222 samples with samples from individuals of a twin pair taken on the same day. These were to be used for the study of biological and technical variability in the various molecular phenotyping platforms, focusing on the unique opportunities afforded by studies in a large set of clinically well characterised monozygotic and dizygotic twins. These studies were all part of the Molecular Phenotyping to Accelerate Genomic Epidemiology (MolPAGE) project. For the MolPAGE project, eligible volunteers were healthy, Caucasian females of North Europe descent, aged between 45-76 years old. The study was approved by St. Thomas' Hospital Research Ethics Committee (EC04/015 Twins UK). The experiments and subsequent analyses presented in this paper are based on serum samples extracted from the fasting blood samples.

### Antibodies, Bead Coupling, Sample preparation and Assay procedure

#### Antibodies

Protocols for antigen selection, cloning, protein expression, immunization of rabbits, and affinity purification to yield polyclonal antibodies were performed as described previously [26,27]. All protein fragments used for immunization were produced with a tag and a target protein part of on average 120 amino acids and in general those proteins had a size of approximately 30 kDa. Antibodies from the Human Protein Atlas (HPA) have been selected as described elsewhere [24] (see also Table 1) and 66 were involved the study, all tested on protein arrays for specificity [28]. In addition rabbit IgG (Jackson ImmunoResearch), a human serum albumin binding protein (Affibody AB) and an anti-IgA antibody (Dako Cytomation) were also included but not highlighted in further detail.

#### Bead coupling

Antibodies were coupled to magnetic carboxylated beads (MagPlex Microspheres, Luminex-Corp.) in accordance to the manufacturer's protocol with minor modifications. For each antibody, 3.2 μg were coupled to 10^6 ^beads in a 96-well half-area microtiter plate (Greiner BioOne) and beads were allowed to sediment for 30 s on a magnetic bead separator (LifeSep™, Dexter Magnetic Technologies Inc.) prior to the removal of supernatants. For an albumin binding Affibody^® ^molecule, 4 μg were coupled. Beads were stored in a protein-containing buffer (Blocking Reagent for ELISA, Roche) with NaN_3_. All coupled beads were re-suspended with sonication in an ultrasonic cleaner (Branson Ultrasonic Corporation) for 5 min prior to storage at 4°C. All antibody-coupled beads were counted using the Luminex LX200 instrument and the coupling efficiency for each antibody was determined via R-Phycoerythrin labeled anti-rabbit IgG antibody (Jackson ImmunoResearch). A bead mixture was created in solution, and optimized as previously described [29].

#### Sample labelling

Protocols for sample labelling and assay procedure were based on earlier work [13]. At first, samples were thawed at room temperature and centrifuged for 10 min at 10,000 rpm, transferred into a microtiter plate (Abgene) and heat treated at 56°C over 30 min in a thermo cycler (Biorad). The plates were centrifuged (1 min at 3,000 rpm) and 3 μl of each sample was added to 24.5 μl 1 × PBS with a liquid handler (Plate mate 2 × 2, Matrix). N-hydroxysuccinimidyl ester of biotinoyl-tetraoxapentadecanoic acid (NHS-PEO4-Biotin, Pierce) was then added at 10-fold molar excess to yield an overall 1/10 sample dilution followed by an incubation over 2 hours at 4°C in a microtiter plate shaker (Thermomixer, Eppendorf). The reaction was stopped by the addition of Tris-HCl, pH 8.0 at a 250-fold molar excess over biotin and incubated for another 20 min at 4°C prior to a final storage at -80°C.

#### Assay procedure

All samples were utilized without removing unincorporated biotin and diluted 1/50 utilizing a liquid handler in a assay buffer composed of 0.5% (w/v) polyvinylalcohol and 0.8% (w/v) polyvinylpyrrolidone (Sigma) in 0.1% casein in PBS (PVXC) supplemented with 0.5 mg/ml non-specific rabbit IgG (Bethyl). A second heat treatment was performed as above and 45 μl sample were added to 5 μl of bead mixtures in a microtiter plate (Greiner BioOne). Incubation took place over night on a shaker at an ambient temperature. Beads were washed in wells with 3 × 50 μl PBST (1 × PBS pH 7.4, 0.1% Tween20) on a magnetic bead separator with a liquid handling system followed 10 min with 50 μl of a stop solution containing 0.1% paraformaldehyde in PBS. Beads were washed 1 × 50 μl PBST and 30 μl of 0.5 μg/ml R-Phycoerythrin labeled streptavidin (Invitrogen) in PBST were added and incubated for 20 min. Finally, beads were washed 3 × 50 μl and measured in 100 μl PBST. Measurements were performed using a Luminex LX200 instrument with Luminex xPONENT software and counting at least 50 events per bead ID, displaying binding events as median fluorescence intensity. A summary of the experimental work flow is presented in Figure 1. In what follows we will refer to each measured proteomic phenotype in the study as an "antibody".

Of the 222 serum samples obtained from fasting blood, 48 samples from 24 MZ twin pairs were split into two aliquots (which usefully allows assessment of technical replicability) rendering an additional 48 sample aliquots. The resulting 270 sample aliquots were then randomized and placed onto three 96-well plates, where each plate contained an extra 6 reference samples. Subsequently a total of 69 antibodies were utilized to render a 270 by 69 matrix of protein (antibody) array data.

### Statistical analysis - Data pre-processing, Modelling and Estimation

#### Data pre-processing

Probabilistic quotient normalization (PQN) [16] was used to pre-process the data. In metabonomics, PQN has been used as a robust method to account for dilution of complex biological mixtures. The method performs well in compensating for different dilutions in samples. We found this normalization method to increase a correlation-based measure of technical reproducibility across two aliquots of the same sample (see Results).

#### Outliers

Intensity distributions of the antibodies after normalization using PQN were investigated to identify samples that were potential outliers. In order to detect potential outliers we used singular value decomposition [17] to project the data into two-dimensional space. Projecting the data onto the space spanned by the first two principal-component loadings vectors is a commonly used technique for detecting anomalous observations whose extreme behaviour explains a considerable proportion of variance in the data.

#### Modelling and Estimation

All subsequent analyses were done on data that had been normalized using PQN and had undergone removal of outlying samples. Further, a Box-Cox transformation [17] was performed on the data from each antibody. This transformed the data so that their distribution more closely resembled a Gaussian, thereby increasing the quality of fit to the data of the Gaussian-based mixed-effects model.

#### Variability analysis

For each molecular phenotype (antibody) we used a statistical model to quantify the biological and experimental variation inherent in the phenotype. As mentioned before, the aim was to partition the total variability in a molecular phenotype into variability that is attributable to familiality (additive genetic, dominant genetic and common environmental), individual environmental, common visit, individual visit and experimental components, respectively. Note that familial and individual environmental variability together make up stable biological variability.

As mentioned previously, the measured quantity of interest was the intensity of fluorescence emitted when an immobilised antibody captured an antigen in a sample. For each antibody let Y_ijkl _denote the normalized and transformed intensity of the *l*th aliquot replicate (1, 2), at the kth visit (1, 2) of the jth twin (1, 2) from the ith twin pair (1, 2, ...,77). The data for each antibody was modelled using a linear mixed-effects model [30-32]:

(1) $${\text{Y}}_{\text{ijkl}}=\mu_{\text{p}\left(\text{i},\text{j},\text{k},\text{l}\right)}+{\text{A}}_{\text{ij}}+{\text{D}}_{\text{ij}}+{\text{C}}_{\text{i}}+{\text{E}}_{\text{ij}}+{\text{W}}_{\text{ik}}+{\text{V}}_{\text{ijk}}+\varepsilon_{\text{ijkl}},$$

where μ_p(i, j, k, l) _is the mean intensity for samples on plate p (the function p(i, j, k, l) maps sample aliquots to plates), A is the additive genetic effect, D is the dominant genetic effect, C is the common environment effect, E is the individual or unique environment effect, W is the common visit effect, V is the individual visit effect, and lastly ε represents the residual experimental effects. The plate mean μ_p(i, j, k, l) _is a fixed effect, while A, D, C, E, W, and V are random effects. It is assumed that the random effects are additive and independent. This implies that the total phenotypic variance can be decomposed as:

$$\text{Var}\left(\text{Y}\right)=\text{Var}\left(\text{A}\right)+\text{Var}\left(\text{D}\right)+\text{Var}\left(\text{C}\right)+\text{Var}\left(\text{E}\right)+\text{Var}\left(\text{W}\right)+\text{Var}\left(\text{V}\right)+\text{Var}\left(\varepsilon \right).$$

The covariance between phenotypic measurements on a pair of samples gathered at the same visit from monozygotic twins is Var(A) + Var(D) + Var(C) + Var(W), and for dizygotic twins is (1/2)Var(A) + (1/4)Var(D) + Var(C) + Var(W). Familiality (fam) is the proportion of variance attributable to genetic and common environmental effects:

$$\text{fam}=\left(\text{Var}\left(\text{A}\right)+\text{Var}\left(\text{C}\right)+\text{Var}\left(\text{D}\right)\right)∕\text{Var}\left(\text{Y}\right).$$

For twin data, the variances Var(A), Var(C) and Var(D) are not all identifiable. However familiality is an estimable function of these variance parameters.

For each antibody, maximum likelihood (ML) estimates of the variance components were obtained by fitting model (1) using the lmer function from the lme4 package in R [33] after normalization, removal of outliers and Box-Cox transformations. The model was reduced prior to fitting. The variances of A, C and D are not identifiable. In order to address non-identifiability we re-parameterized the three non-identifiable variances, Var(A), Var(C) and Var(D) by defining two new random effects H and M (whose variances are identifiable) as follows:

Var(H)=(1∕2)*Var(A)+(1∕4)*Var(D)+Var(C)

Var(M) = (1/2)*Var(A)+(3∕4)*Var(D)

Subsequently the familial variance (which represents the combined effects of genetics and common environment) was obtained as the sum of the estimated variances of H and M, i.e familial variance = Var(H) + Var(M).

Note that if x_1 _and x_2 _are the phenotype measurements of two monozygotic (MZ) twins then the covariance matrix of the measurements is such that: Var(x_1_) = Var(x_2_) = Var(H) + var(M) +Var(E) and covariance(x_1_, x_2_) = Var(H) + Var(M). Similarly if x_1 _and x_2 _are the phenotype measurements of two dizygotic (DZ) twins then covariance matrix of the measurements is such that: Var(x_1_) = Var(x_2_) = Var(H) + Var(M) + Var(E) and covariance(x_1_, x_2_) = Var(H). A similar approach has previously been used and elaborated on metabolomics data [34].

The PQN pre-processing procedure that we applied should mitigate any systematic differences between measurements in the different wells introduced by the instrumental time drift between plate well 1 through to plate well 96. However, in order to investigate if there were any substantial significant intensity-drift effects remaining after PQN we also fit a second model (hereafter referred to as model 2) by introducing a linear, plate-specific well location term into model 1, where well location is a number between 1 and 96 representing the position of a sample in the plate wells. That is in model 2, for each antibody we fit a linear mixed-effects model with linear, plate-specific intensity-drift fixed effects and the random effects A, D, C, E, W, and V as before. Subsequently Akaike's information criterion [35,36] was used to compare the fit of models 1 and 2. Akaike's information criterion (AIC) is a measure of the goodness of fit of an estimated statistical model and is defined as:

AIC=2k - 2ln(L),

where k is the number of parameters in the model, and L is the maximized value of the likelihood function for the estimated model. For a given a data set, several competing models may be ranked according to their AICs and the model with the smallest AIC is chosen as the one that fits the data best. The pre-processed data and R script used to perform the variability analysis can be found as Additional Files 3 and 4.

## Abbreviations

HPA: Human Protein Atlas; IQR: Interquartile range; Fam: familial sources; Env: individual environment effects; Cv: common visit effects; Iv: individual visit effects; Exp: experimental effects; MZ: monozygotic; DZ: dizygotic; PQN: probabilistic quotient normalization; AIC: Akaike's information criterion; LOWESS: locally weighted scatterplot smoothing.

## Competing interests

The authors declare that they have no competing interests.

## Authors' contributions

BSK did the data analysis and generated the manuscript, GN provided guidance on the data analysis and helped generate the manuscript, MN has carried out array experiments and participated in data analysis, MR provided guidance on the data pre-processing and analysis, AB was responsible for storage and distribution of the serum samples, CCH supervised aspects of this manuscript, MU supervised aspects of this manuscript, PN has edited the manuscript and supervised the experiments, TDS supervised aspects of this manuscript, JMS generated the manuscript, designed and carried out array experiments and participated in data analysis. All authors read and approved the final manuscript.

## Appendix

### Correlation model

Consider a proteomics platform, which measures the abundance of *p *proteins, indexed by *k *∈ {1,...,*p*}. Suppose that there are *n *samples, indexed by *i *∈ {1,...,*n*}, and each is measured in technical duplicate, with duplicates indexed by *j *∈ {1, 2}. A model for the observed data is:

$$y_{ij}^{\left(k\right)}=\mu^{\left(k\right)}+\nu_{i}^{\left(k\right)}+\varepsilon_{ij}^{\left(k\right)}$$

where:

• $y_{ij}^{\left(k\right)}$is the abundance of the *k*th protein as measured in the *j*th replicate of the *i*th biological sample

• *μ*^(*k*) ^is the mean (across all samples) of the measured abundance of protein *k*

• $\nu_{i}^{\left(k\right)}$ is the biological deviation in the abundance of protein *k *in sample *i *around *μ*^(*k*)^

• $\varepsilon_{ij}^{\left(k\right)}$ is the experimental variation in replicate *j *of sample *i *at protein *k*.

The correlation-based measure of technical reproducibility used in the current paper was calculated for each protein in turn, as follows. For the *k*th protein, we formed the two vectors $v_{1}^{\left(k\right)}\equiv \left(y_{11}^{\left(k\right)},y_{21}^{\left(k\right)},...,y_{n1}^{\left(k\right)}\right)$ and $v_{2}^{\left(k\right)}\equiv \left(y_{12}^{\left(k\right)},y_{22}^{\left(k\right)},...,y_{n2}^{\left(k\right)}\right)$, and then estimated the correlation between the two: $r^{\left(k\right)}\equiv cor\left(v_{1}^{\left(k\right)},v_{2}^{\left(k\right)}\right)$; *r*^(*k*) ^is directly related to the utility of the platform, as it measures the reproducibility across replicates for each individual protein.

A different, commonly used (and often misinterpreted) measure of correlation would be calculated as follows. For the pair of replicates of sample 1, form the two vectors $w_{11}\equiv \left(y_{11}^{\left(1\right)},y_{11}^{\left(2\right)},...,y_{11}^{\left(p\right)}\right)$ and $w_{12}\equiv \left(y_{12}^{\left(1\right)},y_{12}^{\left(2\right)},...,y_{12}^{\left(p\right)}\right)$, and then estimate the correlation between the two: *s*_1 _≡ *cor*(**w**_11_,**w**_12_). It is less appropriate to use the correlation across proteins, *s*_1_, as this is affected by the overall scale of the range of measurement. For instance, for the same level of technical variation, $Var\left(\varepsilon_{ij}^{\left(k\right)}\right)$, *s*_1 _can be made arbitrarily large by increasing the range between the expected values of the lowest- and highest-expressed protein levels (i.e. increasing the range of the *μ*^(*k*)^).

## Supplementary Material

Additional file 1 **Protein profiles before data processing**. Profiles from all antibodies across all samples are shown before any data processing, with the red line indicating the locally weighted scatterplot smoothing (LOWESS). Click here for file

Additional file 2 **Protein profiles after data processing**. Profiles from all antibodies across all samples are shown after any data processing, with the red line indicating the locally weighted scatterplot smoothing (LOWESS). Click here for file

Additional file 3 **Pre-processed data**. The csv file contains pre-processed intensity values for all antibodies across all samples. Click here for file

Additional file 4 **R script for variability analysis**. The R script can be used to perform the variability analysis with the pre-processed data from Additional File 3. Click here for file
