# Supplementary material for: Variance decomposition of protein profiles from antibody arrays using a longitudinal twin model
Source: Proteome Sci. 2011 Nov 17;9:73. doi: 10.1186/1477-5956-9-73 (PMC3247853; doi:10.1186/1477-5956-9-73)
Supplement: Additional file 1 — Protein profiles before data processing. Profiles from all antibodies across all samples are shown before any data processing, with the red line indicating the locally weighted scatterplot smoothing (LOWESS). [file 1477-5956-9-73-S1.PDF]

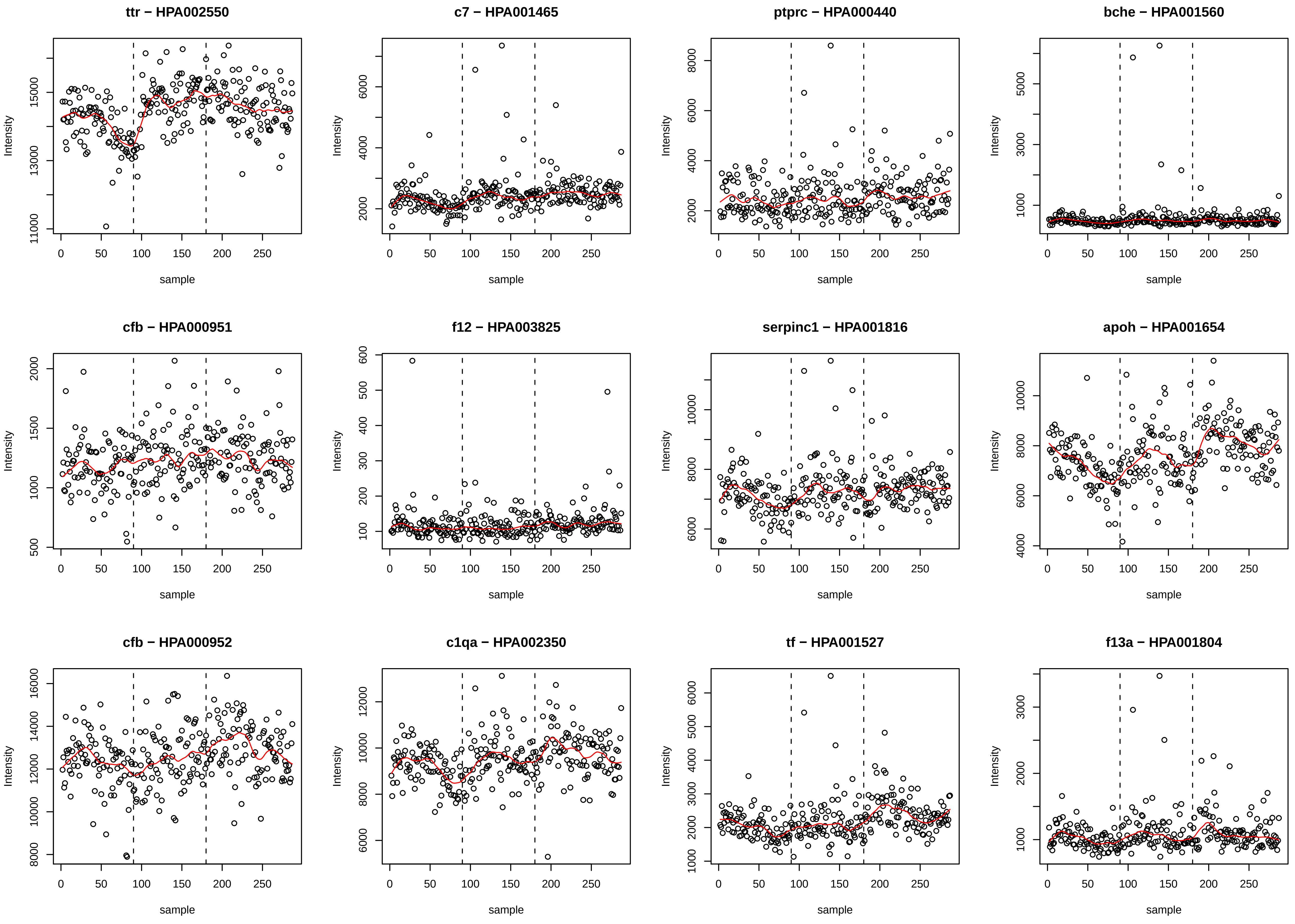
ttr − HPA002550
c7 − HPA001465
ptprc − HPA000440
bche − HPA001560
cfb − HPA000951
f12 − HPA003825
serpinc1 − HPA001816
apoh − HPA001654
cfb − HPA000952
c1qa − HPA002350
tf − HPA001527
f13a − HPA001804
Intensity
sample
0 50 100 150 200 250

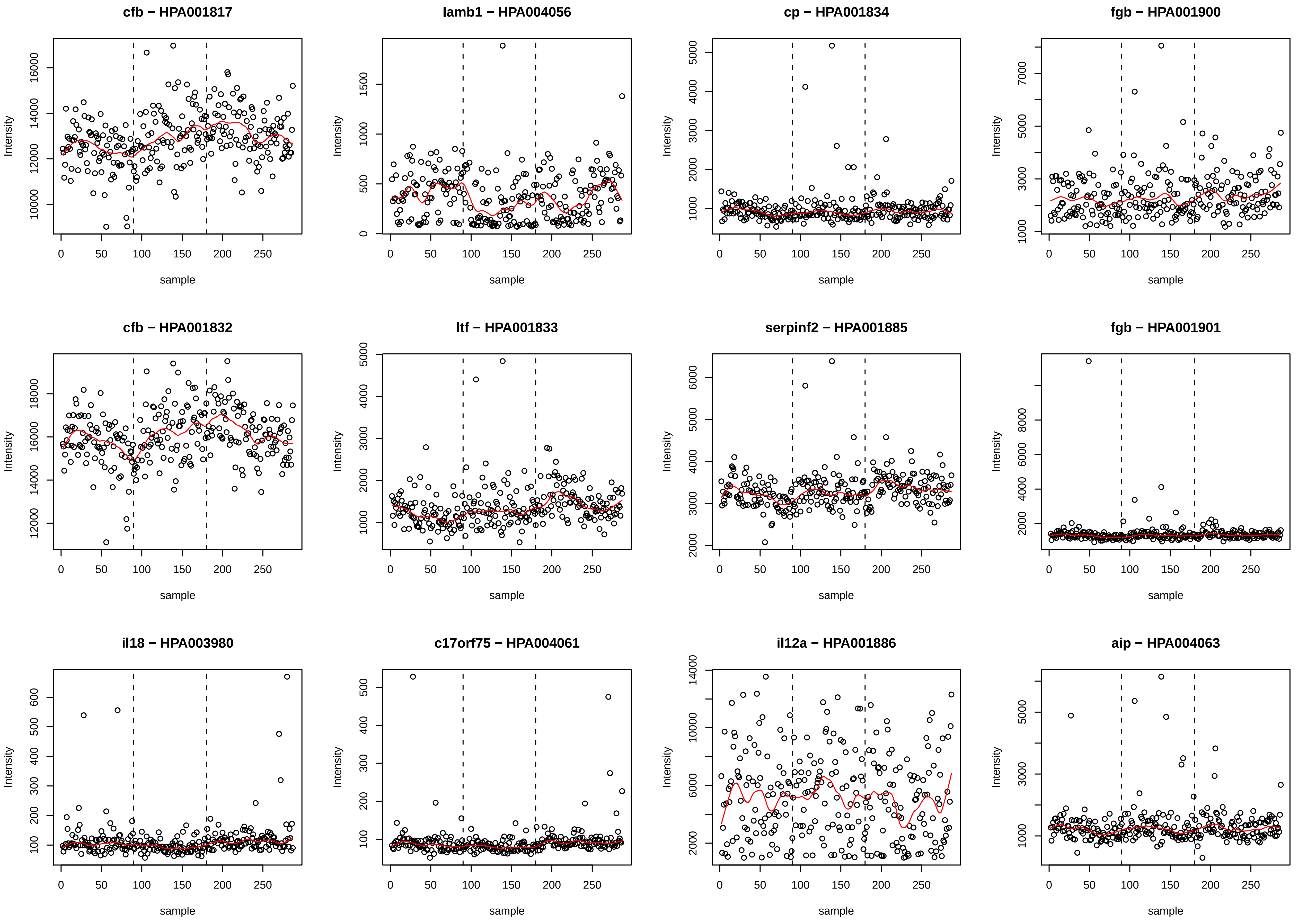
cfb – HPA001817
lamb1 – HPA004056
cp – HPA001834
fgb – HPA001900
cfb – HPA001832
ltf – HPA001833
serpinf2 – HPA001885
fgb – HPA001901
il18 – HPA003980
c17orf75 – HPA004061
il12a – HPA001886
aip – HPA004063
Intensity
sample

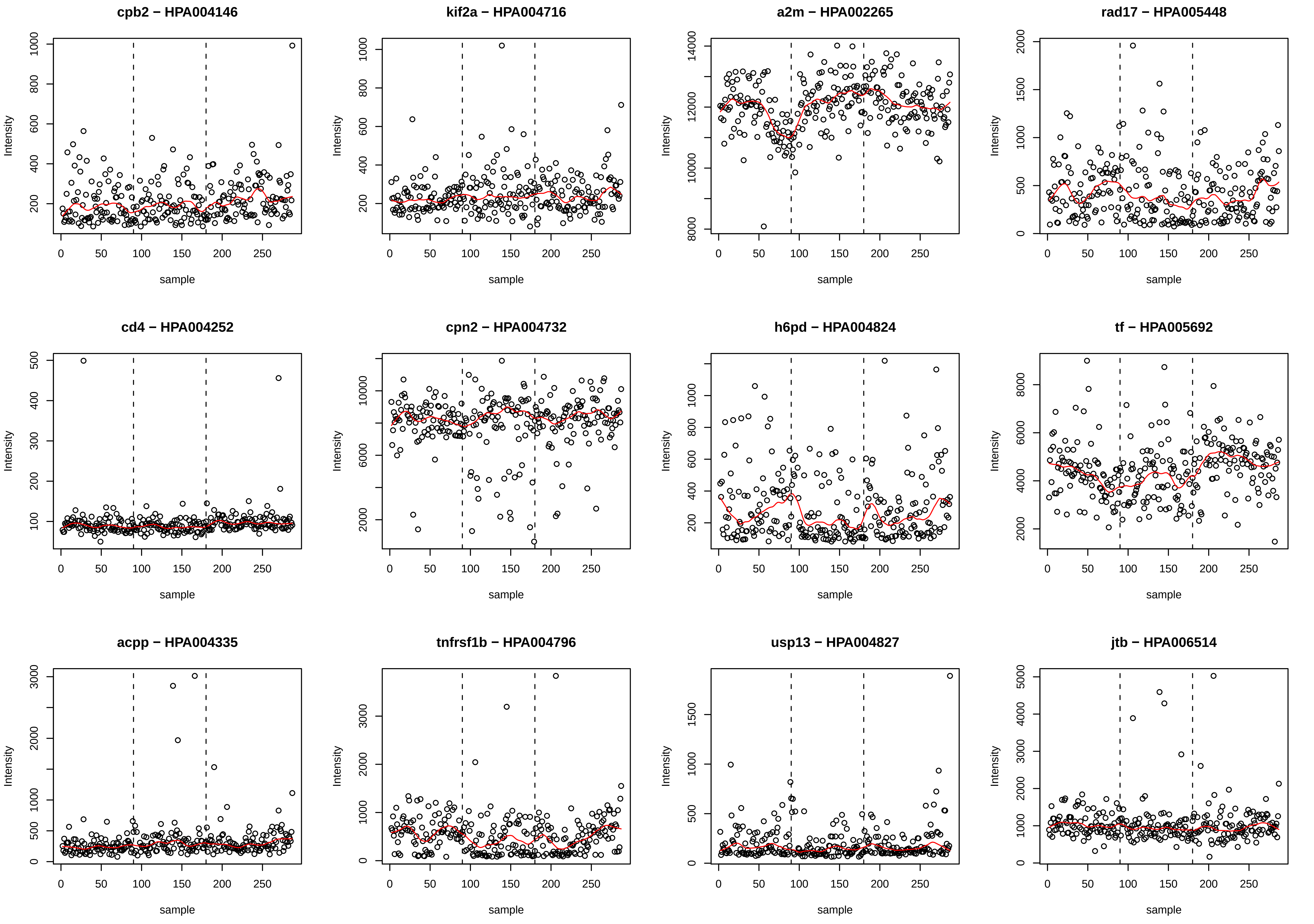
cpb2 − HPA004146
kif2a − HPA004716
a2m − HPA002265
rad17 − HPA005448
cd4 − HPA004252
cpn2 − HPA004732
h6pd − HPA004824
tf − HPA005692
acpp − HPA004335
tnfrsf1b − HPA004796
usp13 − HPA004827
jtb − HPA006514
Intensity
sample

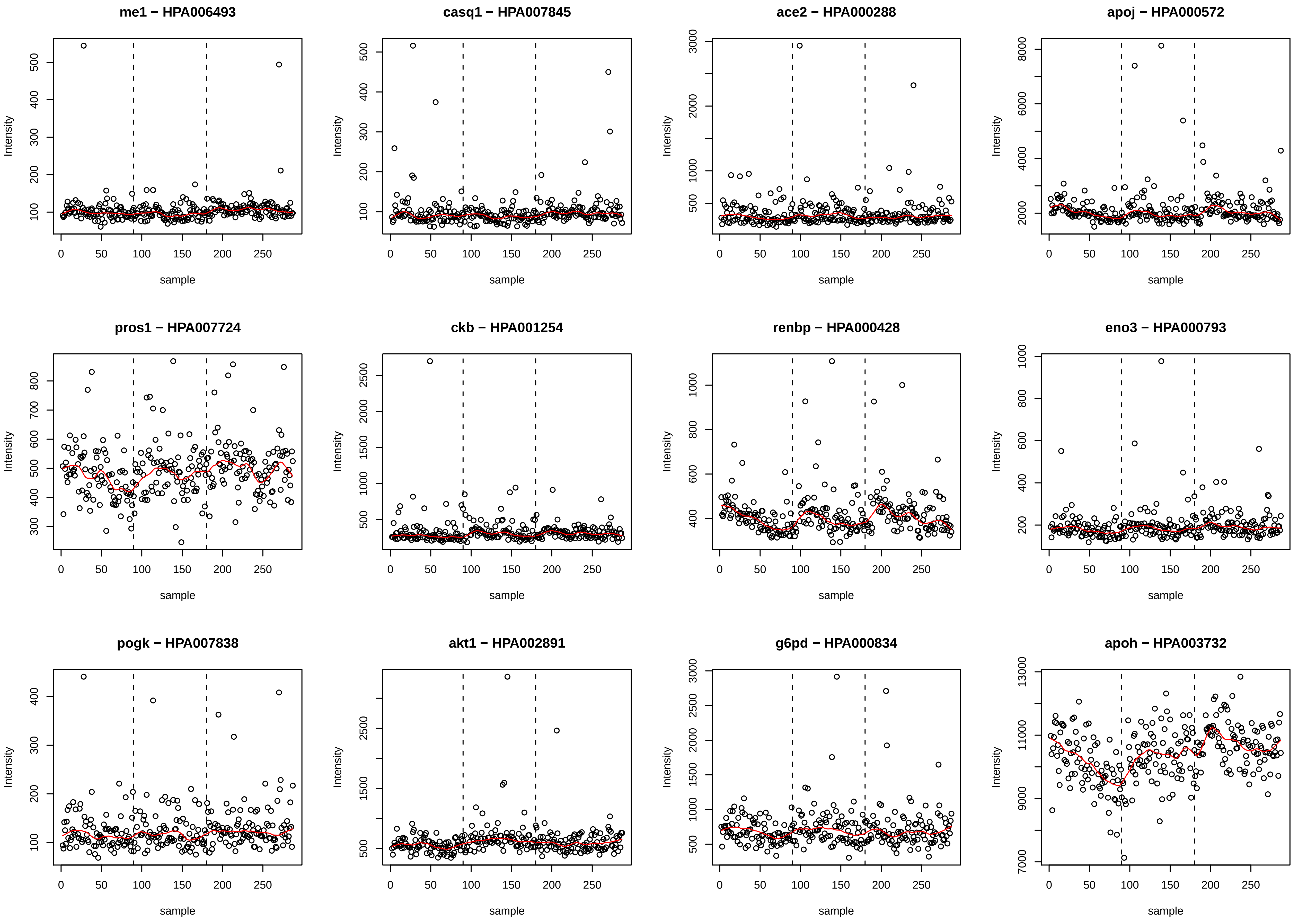

me1 − HPA006493
casq1 − HPA007845
ace2 − HPA000288
apoj − HPA000572
pros1 − HPA007724
ckb − HPA001254
renbp − HPA000428
eno3 − HPA000793
pogk − HPA007838
akt1 − HPA002891
g6pd − HPA000834
apoh − HPA003732
Intensity
sample

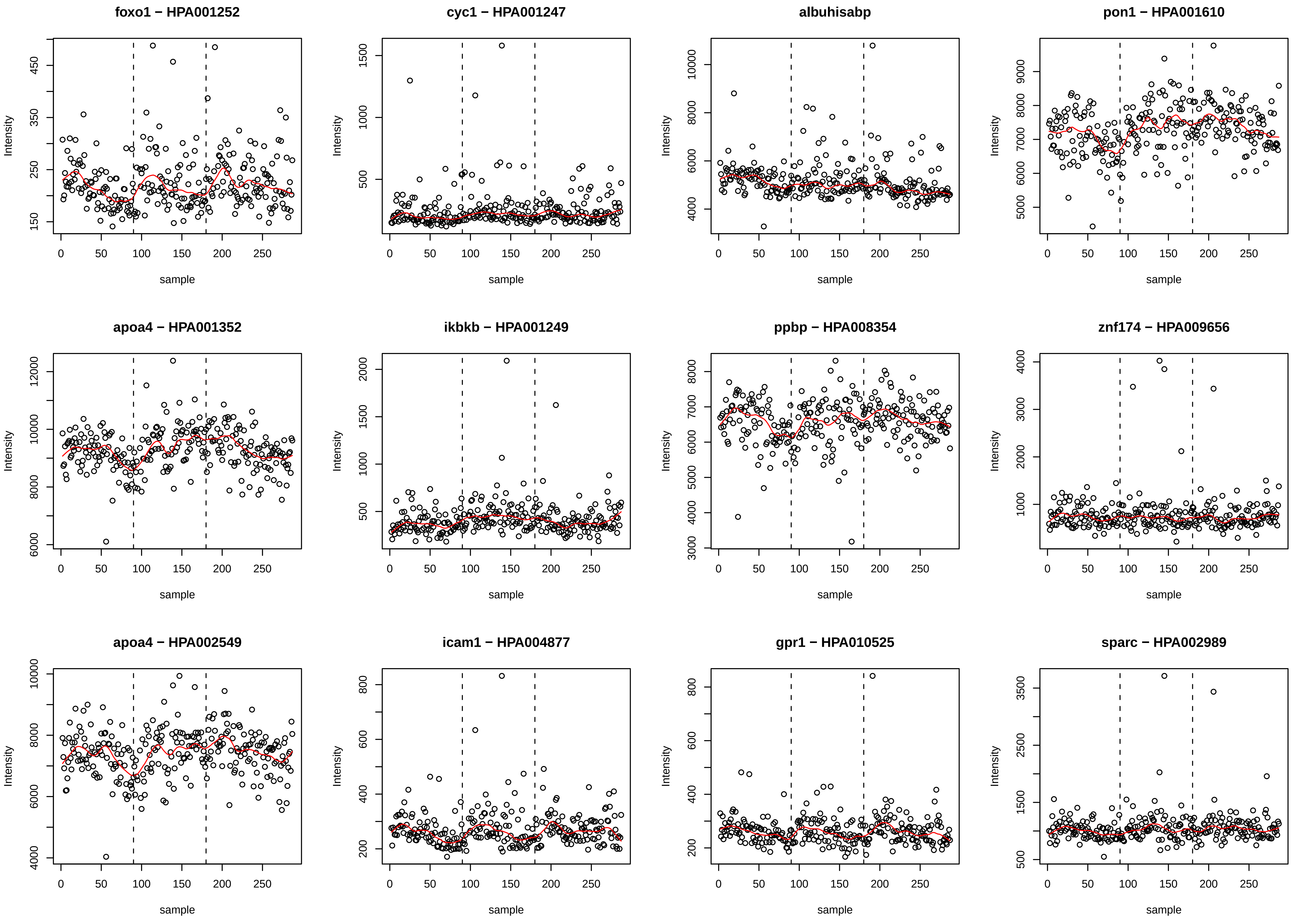
foxo1 – HPA001252
cyc1 – HPA001247
albuhisabp
pon1 – HPA001610
apoa4 – HPA001352
ikbkb – HPA001249
ppbp – HPA008354
znf174 – HPA009656
apoa4 – HPA002549
icam1 – HPA004877
gpr1 – HPA010525
sparc – HPA002989
Intensity
sample

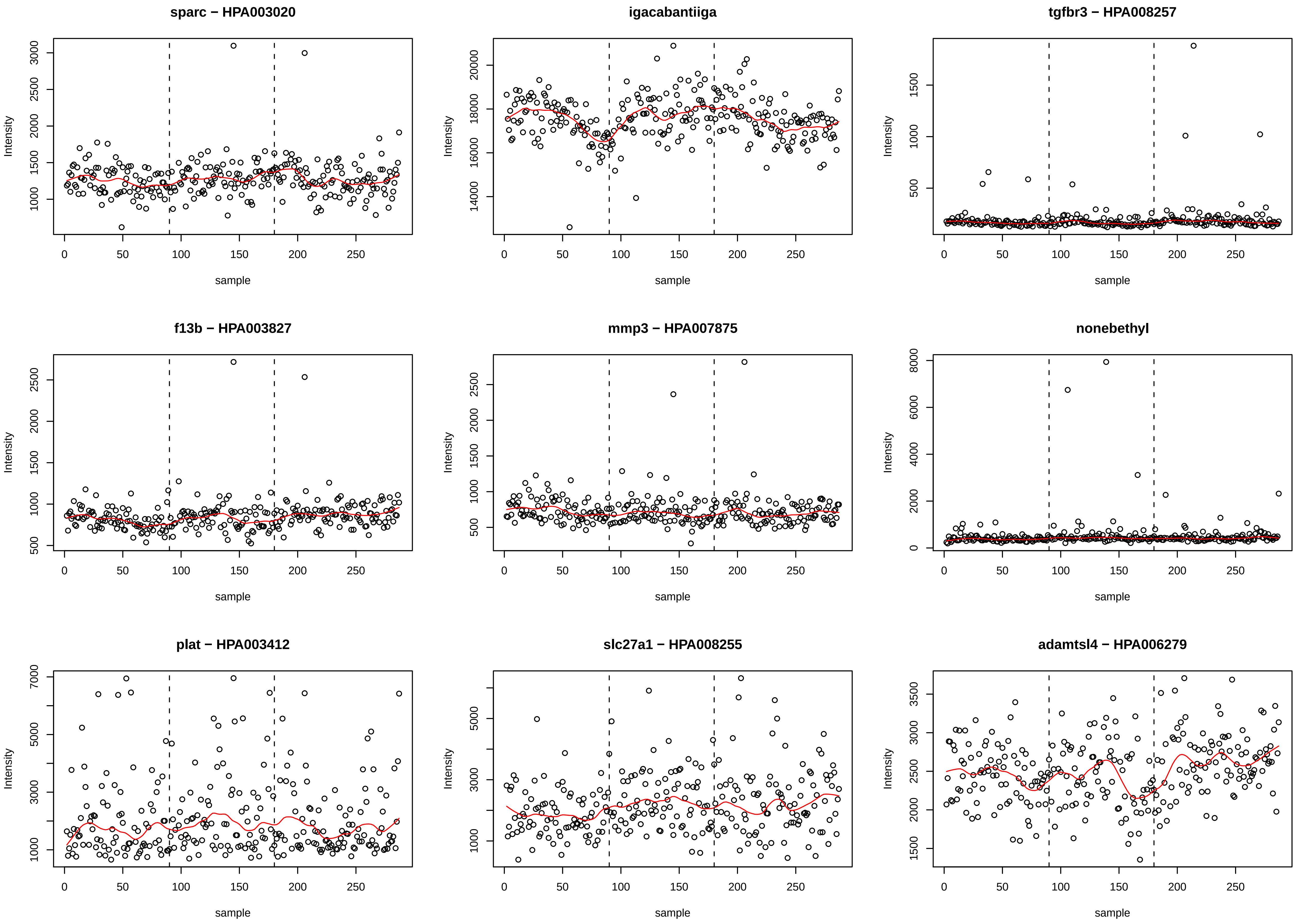
sparc – HPA003020
igacabantiiga
tgfbr3 – HPA008257
f13b – HPA003827
mmp3 – HPA007875
nonebethyl
plat – HPA003412
slc27a1 – HPA008255
adamtsl4 – HPA006279
Intensity
sample
