# Supplementary material for: Variance decomposition of protein profiles from antibody arrays using a longitudinal twin model
Source: Proteome Sci. 2011 Nov 17;9:73. doi: 10.1186/1477-5956-9-73 (PMC3247853; doi:10.1186/1477-5956-9-73)
Supplement: Additional file 2 — Protein profiles after data processing. Profiles from all antibodies across all samples are shown after any data processing, with the red line indicating the locally weighted scatterplot smoothing (LOWESS). [file 1477-5956-9-73-S2.PDF]

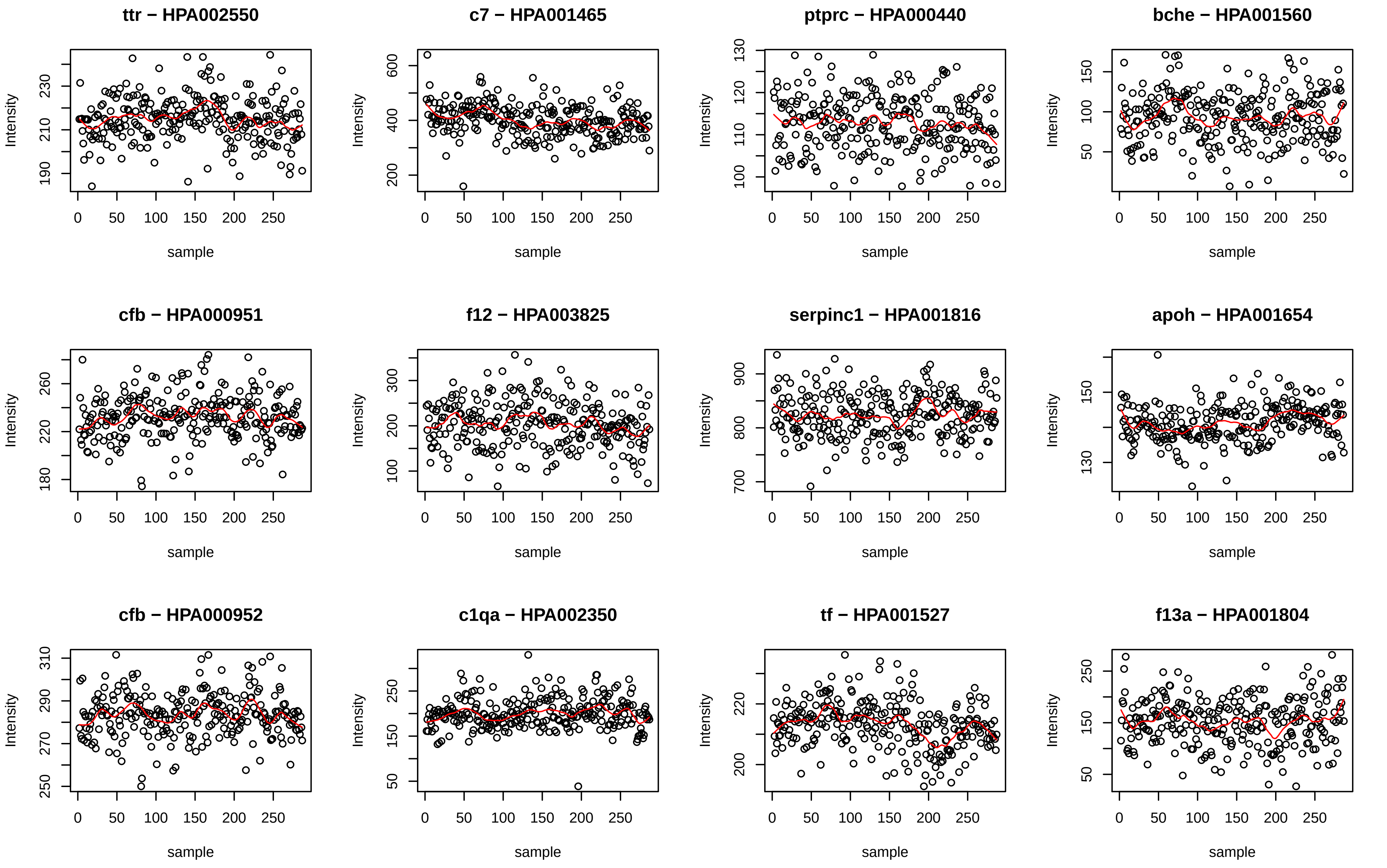
ttr − HPA002550
c7 − HPA001465
ptprc − HPA000440
bche − HPA001560
cfb − HPA000951
f12 − HPA003825
serpinc1 − HPA001816
apoh − HPA001654
cfb − HPA000952
c1qa − HPA002350
tf − HPA001527
f13a − HPA001804
Intensity
sample

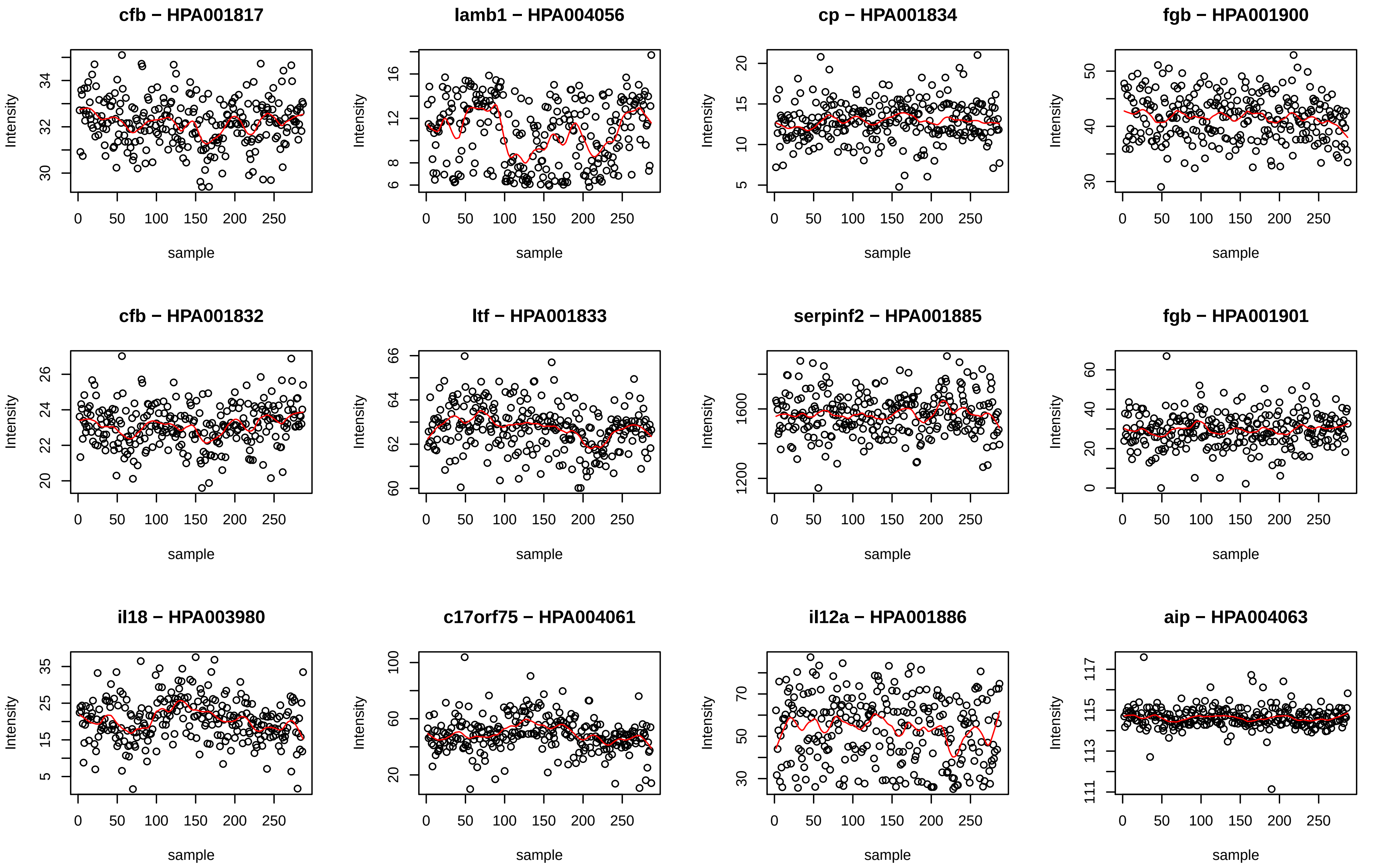
cfb − HPA001817
lamb1 − HPA004056
cp − HPA001834
fgb − HPA001900
cfb − HPA001832
ltf − HPA001833
serpinf2 − HPA001885
fgb − HPA001901
il18 − HPA003980
c17orf75 − HPA004061
il12a − HPA001886
aip − HPA004063
Intensity
sample
0 50 100 150 200 250

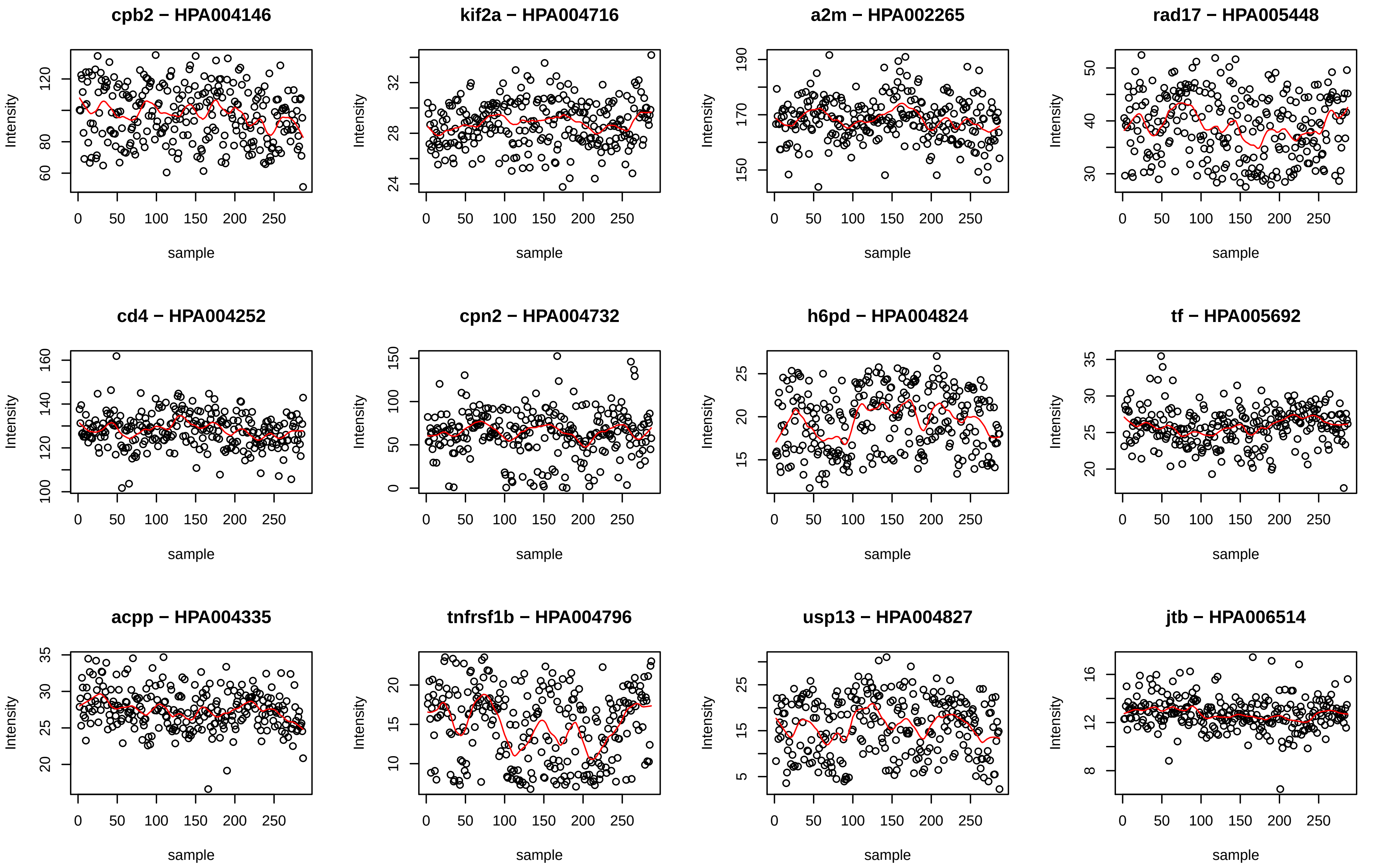
cpb2 − HPA004146
kif2a − HPA004716
a2m − HPA002265
rad17 − HPA005448
cd4 − HPA004252
cpn2 − HPA004732
h6pd − HPA004824
tf − HPA005692
acpp − HPA004335
tnfrsf1b − HPA004796
usp13 − HPA004827
jtb − HPA006514
Intensity
sample
0 50 100 150 200 250

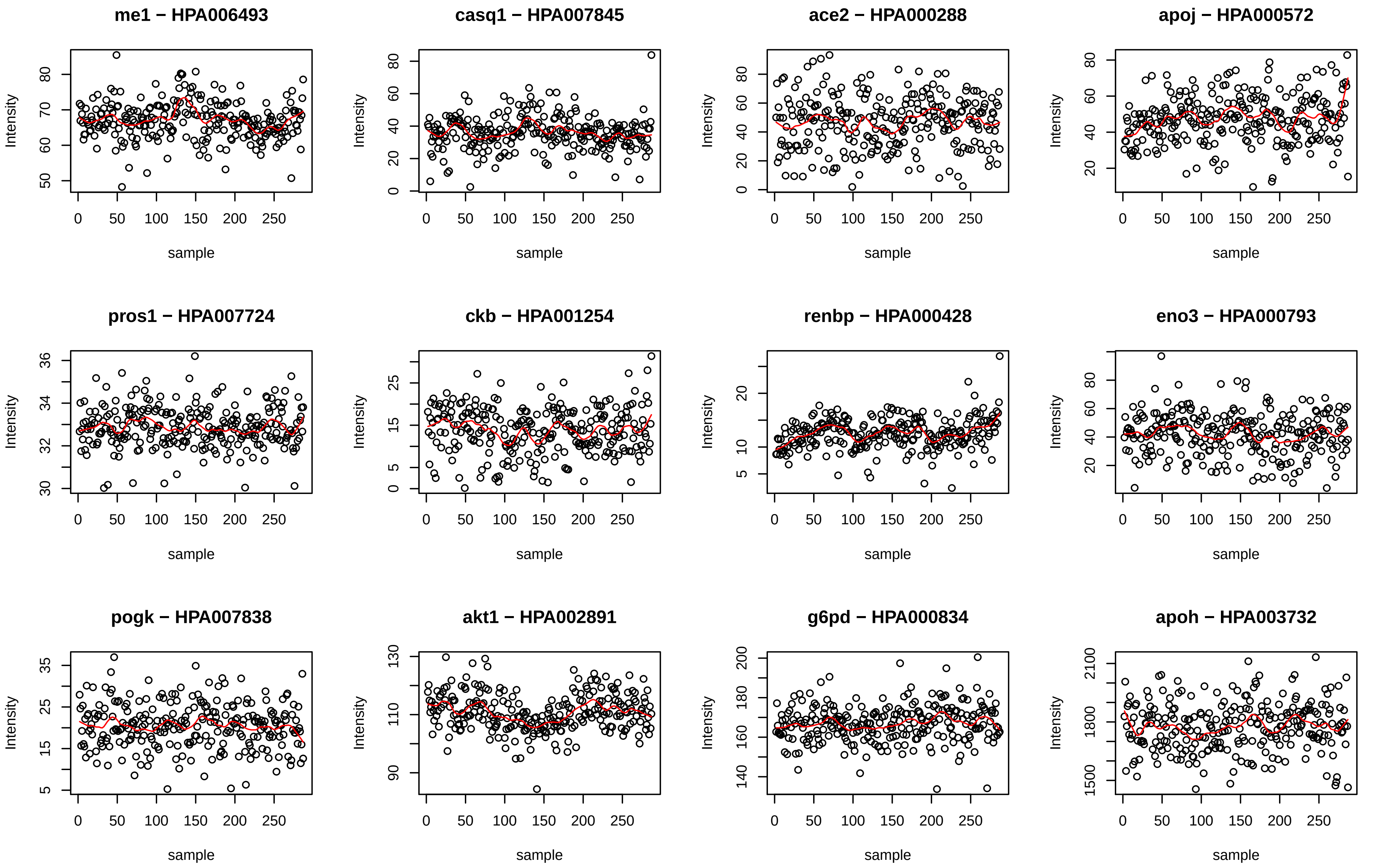
me1 − HPA006493
casq1 − HPA007845
ace2 − HPA000288
apoj − HPA000572
pros1 − HPA007724
ckb − HPA001254
renbp − HPA000428
eno3 − HPA000793
pogk − HPA007838
akt1 − HPA002891
g6pd − HPA000834
apoh − HPA003732
Intensity
sample

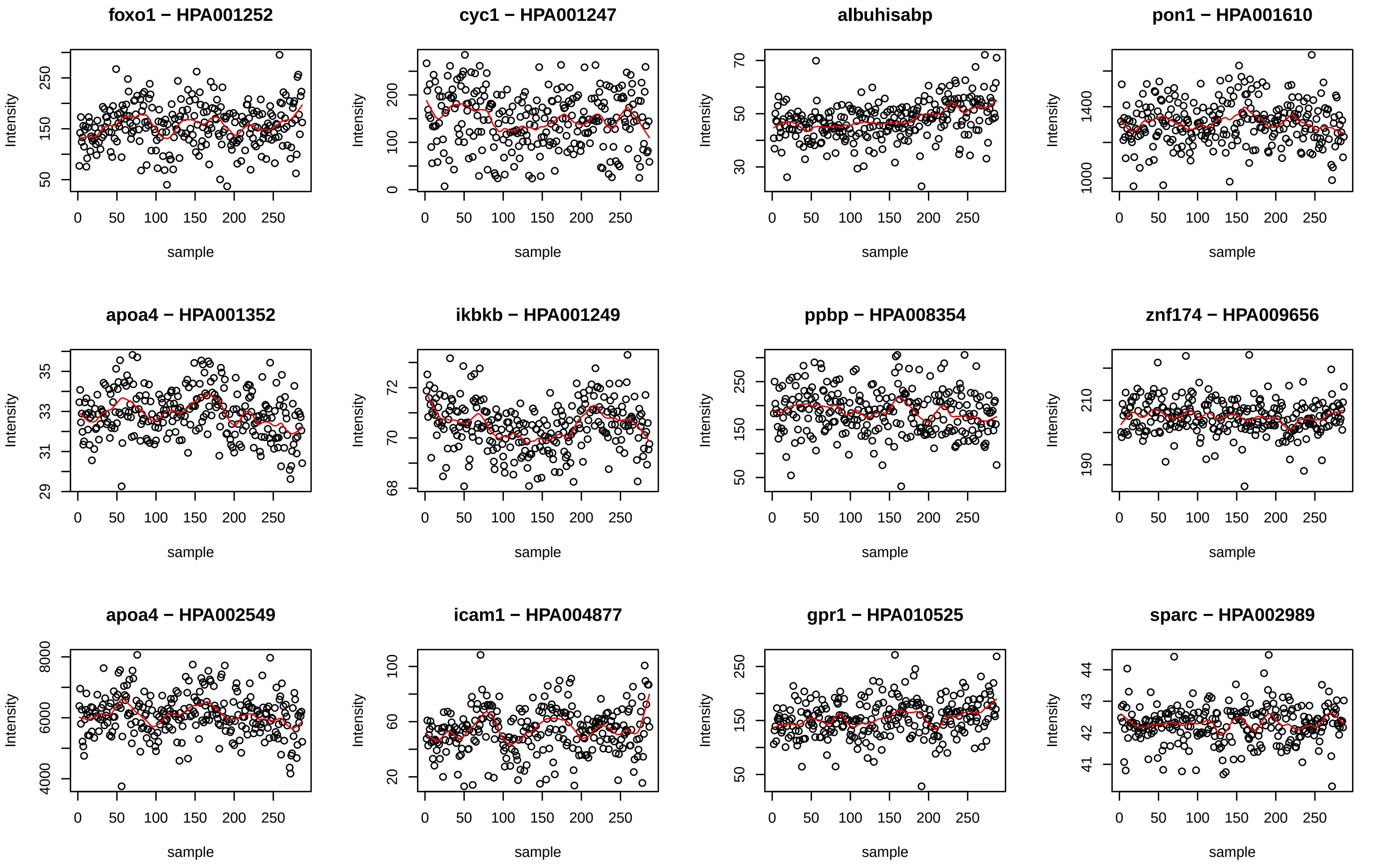
foxo1 − HPA001252
cyc1 − HPA001247
albuhisabp
pon1 − HPA001610
apoa4 − HPA001352
ikbkb − HPA001249
ppbp − HPA008354
znf174 − HPA009656
apoa4 − HPA002549
icam1 − HPA004877
gpr1 − HPA010525
sparc − HPA002989
Intensity
sample

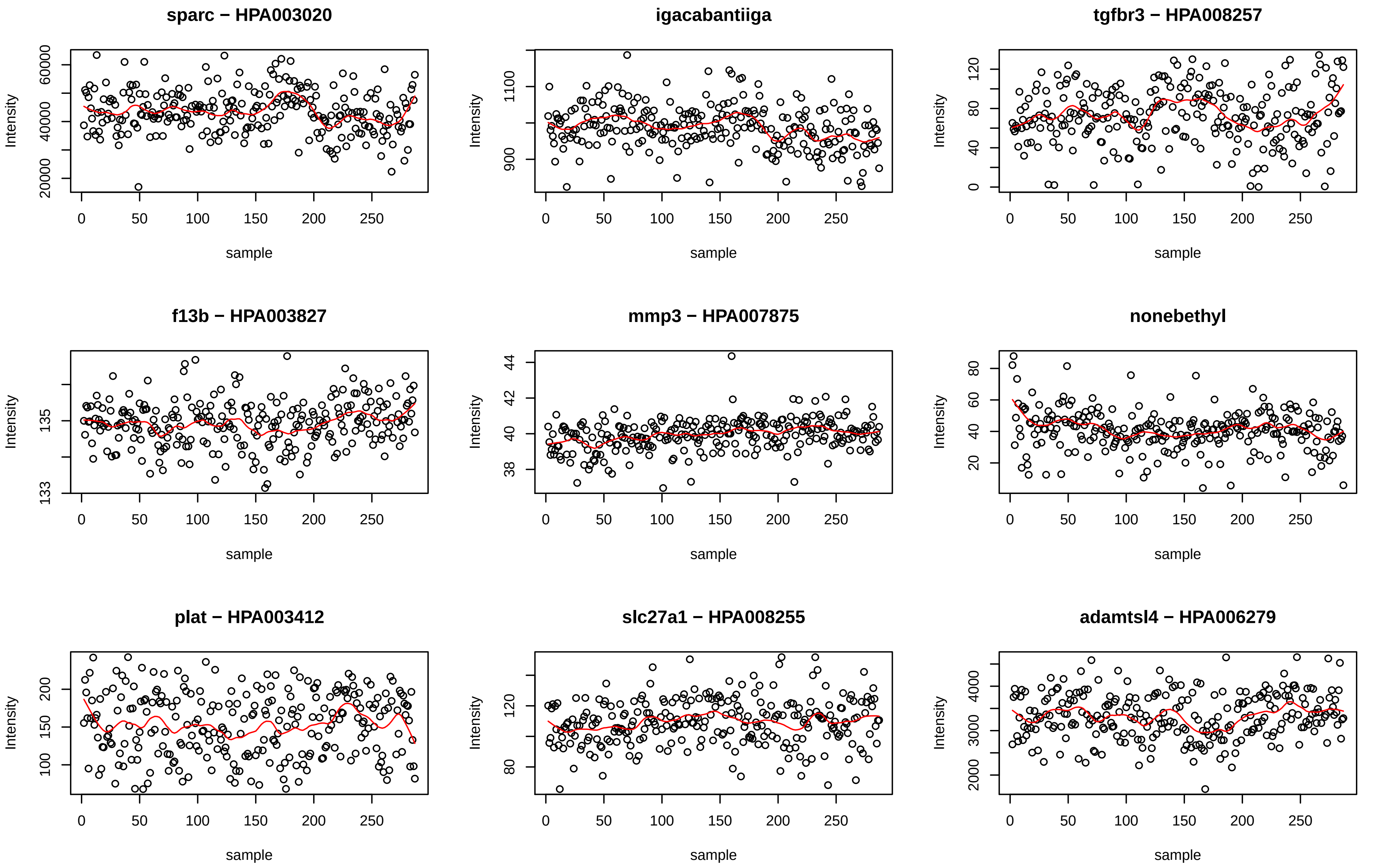

sparc − HPA003020
Intensity
20000
40000
60000
sample
0
50
100
150
200
250
igacabantiiga
Intensity
900
1100
sample
0
50
100
150
200
250
tgfbr3 − HPA008257
Intensity
0
40
80
120
sample
0
50
100
150
200
250
f13b − HPA003827
Intensity
133
135
sample
0
50
100
150
200
250
mmp3 − HPA007875
Intensity
38
40
42
44
sample
0
50
100
150
200
250
nonebethyl
Intensity
20
40
60
80
sample
0
50
100
150
200
250
plat − HPA003412
Intensity
100
150
200
sample
0
50
100
150
200
250
slc27a1 − HPA008255
Intensity
80
120
sample
0
50
100
150
200
250
adamtsl4 − HPA006279
Intensity
2000
3000
4000
sample
0
50
100
150
200
250
